# Comparison of social structures within cities of very different sizes

**DOI:** 10.1098/rsos.150526

**Published:** 2016-02-24

**Authors:** P. Grindrod, T. E. Lee

**Affiliations:** Mathematical Institute, University of Oxford, Oxford OX2 6GG, UK

**Keywords:** social networks, modularity, scaling

## Abstract

People make a city, making each city as unique as the combination of its inhabitants. However, some cities are similar and some cities are inimitable. We examine the social structure of 10 different cities using Twitter data. Each city is decomposed to its communities. We show that in many cases one city can be thought of as an amalgamation of communities from another city. For example, we find the social network of Manchester is very similar to the social network of a virtual city of the same size, where the virtual city is composed of communities from the Bristol network. However, we cannot create Bristol from Manchester since Bristol contains communities with a social structure that are not present in Manchester. Some cities, such as Leeds, are outliers. That is, Leeds contains a particularly wide range of communities, meaning we cannot build a similar city from communities outside of Leeds. Comparing communities from different cities, and building virtual cities that are comparable to real cities, is a novel approach to understand social networks. This has implications when using social media to inform or advise residents of a city.

## 1. Introduction

Cities are often compared to organisms, growing in size and complexity and attracting innovation and economic progress. Yet, despite a century of effort, our understanding of how cities evolve is still woefully inadequate [1]. Beneath the apparent chaos and diversity, there is strong order and a pattern that emerges from the myriad of decisions and processes required for a city to develop and expand physically [2] and socially.

A city has many networks, for example the road, the gas, the electric and the main drain networks. However when we think of the character of a city, these physical networks are unlikely to be our immediate thought. Instead we characterize a city by the people within the city, and the social network that they form—the essence of any society. Despite previous fears that cities impair communities [3], the rise of social media confirms that communities are very much present in urban life.

One can think of each city as a unique amalgamation of social communities that differentiate it from other cities. But how unique is each city? Would an amalgamation of social communities from one city resemble another city? This paper resamples the communities from one city to create virtual cities of any size. By comparing these virtual cities to real cities of a corresponding size, we establish that many cities do not conflict with the proposition that they are indeed made up of the same social building blocks. Alternatively, we may identify which cities are inimitable.

Insight into the social structure of cities may be used to ensure success of media campaigns, such as those which aim to bring social cohesion or to inform, or other types of behavioural intervention. If a social campaign was successful in one city, it is also likely to be successful in cities comprising similar social building blocks. Moreover, it may prove economical to test a campaign in a smaller city before transposing the learnings gained to a larger city.

Generally physical networks evolve alongside the development of a city. Cities develop from the bottom up, and grow to facilitate a division of labour that generates scale economies [4]. This gives rise to a power-law which has been used to predict many factors for a city, from its infrastructure [5], to its carbon footprint [6,7], to patenting activity [5]. Additionally, Batty [1] considers the scaling laws of cities and shows that as cities grow in size, they change in shape through allometry.

Size is the major determinant of most characteristics of a typical city; history, geography and design have secondary roles [8,9]. Using scaling arguments, we show how a social network of a city may follow power-law growth (as in [5–7]), or become saturated (as in [1]).

Community structure within communities has been studied for a long time (e.g. [10]), yet there is still little understood about what to do with the communities once they have been detected [11]. Furthermore, how the interaction in small groups aggregates to form large-scale patterns eludes us in most cases [12].

While a social community may be relatively simple to identify in a social context, there are many different heuristics to formally identify communities. Ideally, each community is strongly connected, and connections between communities are few. However, methods can differ in what is deemed ‘strongly connected’. A review article by Lancichinetti & Fortunato [13] discusses many different methods. Porter *et al.* [11] describe the most popular methods, and apply them to real-life examples. A thorough comparison of different methods’ performance, in terms of computation time and output, is provided by Danon *et al.* [14]. Research on identifying communities is ongoing—inferring agreement that networks can be considered as a collection of communities, despite disagreement as to how to identify them. The work presented here will hold true independent of the method chosen to identify the communities.

Our treatment assumes that the modularity of a given network may be calculated by some ready means (usually accessible within standard routines based on chosen heuristics). We focus on the definition of new networks as sparsely coupled-up unions of separate ‘sub-community’ networks. In doing so, we derive a useful lower bound on the overall modularity by combining some properties (including the modularity) of each of the component sub-communities determined in isolation, see §2. Therefore, a lower bound for the modularity for a large network can be represented as a combination of the modularities of the communities within the network. This relationship is verified by building virtual cities from multiple Erdos–Rényi graphs, see §3. Although we can choose Erdos–Rényi graphs to ensure similar communities (that is, communities with same number of nodes and the same probability of connection between any two nodes), data from real cities may show more variance across communities. We use social networks from 10 different UK cities to examine the relationship between the modularity of the communities and the modularity of the city, see §4. Next we establish whether one city can be represented as a collection of communities from another city, where a city is defined by modularity of the social network, see §5. We discuss our findings in §6.

## 2. Modularity for unions of networks

The modularity *Q*∈[0,1] of a graph defines how easily a graph can be separated into communities, where each node belongs to only one community. A modularity close to 1 indicates clear community structure, where as modularity close to 0 indicates a complete graph, which cannot be divided into communities. The authors are unaware of any work which relates the modularity of the communities of a network to the modularity of the network as a whole.

Consider a set of *R*≥1 separate networks, say {*G*^(*s*)^|*s*=1,…,*R*}. Each graph, *G*^(*s*)^, has exactly *n*_*s*_ vertices and exactly *m*_*s*_ edges; and has modularity *Q*_*s*_ that is subordinate to an optimal partition of the vertices of *G*^(*s*)^ into communities. By considering the *G*^(*s*)^ as communities which make up a network, we show that the modularity of the larger network is a function of the modularity of the communities *Q*_*s*_. This greatly reduces the size of the problem when deriving the modularity of a graph.

Consider a set of *R*≥1 separate networks, say {*G*^(*s*)^|*s*=1,…,*R*}. Each graph, *G*^(*s*)^, has exactly *n*_*s*_ vertices and exactly *m*_*s*_ edges; and has modularity *Q*_*s*_ that is subordinate to an optimal partition of the vertices of *G*^(*s*)^ into communities. In the usual notation [15], with a double sum over all pairs of vertices, we have

$$Q_{s}=\frac{1}{2m_{s}}\sum_{i,j}\left(A_{ij}^{\left(s\right)}-\frac{k_{i}^{\left(s\right)}k_{j}^{\left(s\right)}}{2m_{s}}\right)\delta \left(C_{i}^{\left(s\right)},C_{j}^{\left(s\right)}\right).$$

Here *A*^(*s*)^ is the *n*_*s*_×*n*_*s*_ adjacency matrix for *G*^(*s*)^; *k*_*i*_ is the degree of the *i*th vertex in *G*^(*s*)^; $C_{i}^{\left(s\right)}$ denotes the unique community within *G*^(*s*)^ that contains vertex *i*, and $\delta (C_{i}^{\left(s\right)},C_{j}^{\left(s\right)})=1$ if $C_{i}^{\left(s\right)}=C_{j}^{\left(s\right)}$, and zero otherwise. Thus, $F_{s}=\sum_{i,j}A_{ij}^{\left(s\right)}\delta (C_{i}^{\left(s\right)},C_{j}^{\left(s\right)})$ is the number edges that remain within a single community (0≤*F*_*s*_≤*m*_*s*_) and we may write this as

$$Q_{s}=\frac{F_{s}}{2m_{s}}-\frac{K_{s}}{{\left(2m_{s}\right)}^{2}},$$

where we define $K_{s}=\sum_{i,j}k_{i}^{\left(s\right)}k_{j}^{\left(s\right)}\delta (C_{i}^{\left(s\right)},C_{j}^{\left(s\right)})$.

Now suppose that we consider the whole set of networks as single network, $G=\cup_{s=1}^{R}G^{\left(s\right)}$. Then *G* has exactly $n=\sum_{s=1}^{R}n_{s}$ vertices and exactly $m=\sum_{s=1}^{R}m_{s}$ edges. The modularity of *G*, *Q*, must satisfy

$$Q\geq \frac{1}{2m}\sum_{s=1}^{R}\sum_{i,j}\left(A_{ij}^{\left(s\right)}-\frac{k_{i}^{\left(s\right)}k_{j}^{\left(s\right)}}{2m}\right)\delta \left(C_{i}^{\left(s\right)},C_{j}^{\left(s\right)}\right),$$

since we may use the union individual partitions for the *G*^(*s*)^ to define a partition for *G*. Note that we must use the total number of edges *m* within the *objective* on the right-hand side that is to be maximized in order to define *Q*.

Now rewriting this, we have

$$2\text{mQ}\geq \sum_{s=1}^{R}F_{s}-\frac{K_{s}}{\left(2m\right)},$$

and substituting for *F*_*s*_ we have

$$2\text{mQ}\geq \sum_{s=1}^{R}2m_{s}Q_{s}+\sum_{s=1}^{R}K_{s}\left(\frac{1}{2m_{s}}-\frac{1}{2m}\right).$$

Thus,

2.1 $$Q\geq \sum_{s=1}^{R}\frac{m_{s}}{m}Q_{s}+\frac{1}{4m^{2}}\sum_{s=1}^{R}\frac{m-m_{s}}{m_{s}}K_{s}.$$

Equivalently, we have defined an upper bound on the modularity of *Q*.

2.2 $$Q\geq \frac{1}{m^{2}}\sum_{s=1}^{R}m_{s}^{2}Q_{s}+\sum_{s=1}^{R}\frac{m_{s}}{m}\frac{\left(m-m_{s}\right)}{m}\left(\frac{F_{s}}{2m_{s}}\right).$$

Even if we add or rewire a very small number of edges (relative to each *m*_*s*_) within *G*, thereby connecting up some communities across the *G*^(*s*)^, analogous to a small world construction, we could expect this estimated lower bound for *Q* to remain valid. This is because the vast majority of the edges would remain within communities while the individual partitions maximize the individual modularities.

Modularity lacks sensitivity near the optimal (e.g. figure 3). Therefore, there may be good reason for assuming equality within equations (2.1) and (2.2), since the partitions of the *G*^(*s*)^ are optimal for each and might identify very densely connected subsets of vertices, which would remain so in the union.

For example, suppose each *G*^(*s*)^ is a clique, and there are few edges connecting cliques to each other. And further suppose that each clique of a similar size, $m_{s}=\bar{m}$ say. Then each *F*_*s*_/2*m*_*s*_ is very close to unity, and *Q*_*s*_ is close to zero for a clique (the modularity for a graph consisting of a single clique-community). Thus, equation (2.2) implies

2.3 $$Q_{s}=1-\frac{1}{R}.$$

More generally, suppose that each of graphs *G*^(*s*)^ has the same number of edges, $m_{s}=\bar{m}$; the same intra-community edge density, $(F_{s}/2m_{s})=\bar{\rho }$; and the same modularity, $Q_{s}=\bar{Q}$. Assuming equality in equation (2.2),

2.4 $$Q=\frac{\bar{Q}}{R}+\left(1-\frac{1}{R}\right)\bar{\rho },$$

which generalizes equation (2.3). Since by definition $\bar{\rho }=(F_{s}/2m_{s})>\bar{Q}$ then *Q*, given by equation (2.4), is an increasing function of *R*.

We might have deduced the form of equation (2.4) directly by scaling arguments. If we write $Q=H(\bar{Q},R)$ for some function *H*, and if *R* is split into two factors, *R*=*R*_*a*_*R*_*b*_, then we see immediately that *H* must satisfy

2.5 $$H(\bar{Q},R_{a}R_{b})=H(H(\bar{Q},R_{a}),R_{b}),\;H(\bar{Q},1)=\bar{Q}\;\mathrm{and}\;H\in [0,1].$$

This has solutions $H(\bar{Q},R)=\bar{Q}R^{\alpha }+\beta (1-R^{\alpha })$ for constants *α* and *β*∈[0,1] (see appendix A). When *α*>0 we have growth which follows a power law, and when *α*<0 the system becomes saturated.

Equation (2.4) is a good estimate of the modularity of network as a function of the modularities of its communities. Since modularity is an *NP* hard problem, there may be circumstances where it is simpler to calculate the modularity of numerous smaller networks than one large network. We now use Erdos–Rényi graphs to test the lower bound (equation (2.4)).

## 3. Erdos–Rényi graphs

Let us assume that each *G*^(*s*)^ is an Erdos–Rényi graph over *n*_*s*_ vertices independently drawn from ER(*n*_*s*_,*p*), for some probability *p*. Then *G* is just the union of these relatively highly connected ‘ghettos’ (the *G*^(*s*)^). If we wish we could also add a very small number of edges so as to connect up a few vertices within the separate ghettos. Such a ghetto-model gives rise to a joint distribution for the triple (*m*_*s*_,*Q*_*s*_,*F*_*s*_). Suppose we generate *R* such graphs and calculate the modularity *Q* of the union *G*, using a chosen standard method, in this case one based on the successive bisection of communities [15,16], as implemented in Mathematica (see next section).

**Figure 1.**
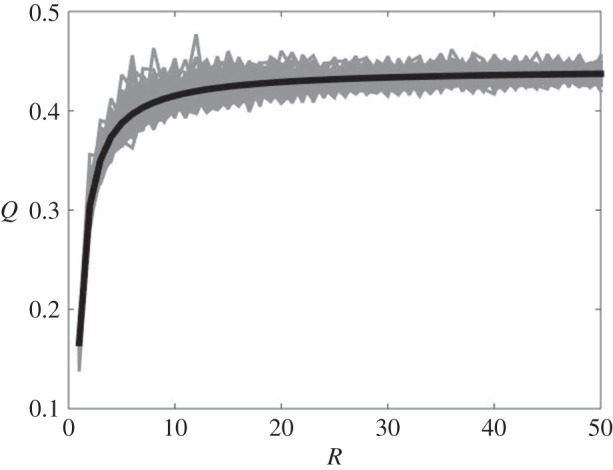
Growing virtual cities, by successively increasing *R* and adding further ghettos *G*^(*s*)^∼ER(100,0.2), and contrasting with the estimate from equation (2.4) (black solid curve).

Then as we increase *R* we may observe how *Q* increases. In fact we may generate an ensemble of such increasing virtual cities *G*, made up from *R* independent ghetto graphs, *G*^(*s*)^, drawn from ER(*n*,*p*). We may contrast equation (2.4), by plugging in corresponding expected values for $\left(\bar{Q},\bar{\rho }\right)$, with the simulated envelope of *Q* values observed. In figure 1, we chose *n*_*s*_=100, for each *s*=1,…,*R*, and *p*=0.2; and drew each *G*^(*s*)^ independently from ER(100,*p*). The envelope is at its widest at small and intermediate values where *R*<20 and is not so large as to provide a reliable sampling of the distribution assumed for the ghettos. For larger *R*, we see convergence of *Q* towards $\bar{\rho }>\bar{Q}$. This is where the sums in equation (2.2) are very well represented by sums over *R* copies of the averaged value for the *s*-dependent terms (from the joint distribution for the triple (*m*_*s*_,*Q*_*s*_,*F*_*s*_)).

After introducing some suitable dataset representing the digital social networks within distinct British cities in the next section, we shall apply the theory developed in this section. By using the community sub-structure observed from any particular city, City *A*, we may thus generate other *virtual cities* (by sampling from *A*’s communities with repetition) that are made of the *same stuff*, the same underlying distribution of component communities as City *A*, yet are of any size we chose. In particular, we may match the size of any other observed city, City *B*. So we shall be able to ask whether City *B* is similar or not to one made of the same underlying components as City *A*, while allowing for their differences in scale.

## 4. Reciprocated mentions networks

We defined a reciprocated (Twitter) mentions network, over 10 UK cities and a four week period, as follows.

We identified all those Twitter accounts that were active during 1–28 October 2014 (four full weeks), having biographical information indicating that they are associated with one of 10 cities: London, Manchester, Leeds, Sheffield, Nottingham, Birmingham, Bristol, Cardiff, Glasgow and Edinburgh. The data were collected, using the DataSift Twitter Firehose access, by selecting individuals who had one of the 10 cities stated in their user description field. (We did not look for geo-coded Tweets because the coverage would be too sparse for it to be useful.) We trapped all Tweets during that period (excluding all ReTweets, which accounted for around one-third of all Tweets). This resulted in 14 946 171 original Tweets made by 321 255 individual accounts.

The giant components from using 14 days of data are not sufficiently different from the corresponding giant components using 28 days of data, and a diminishing returns effect becomes apparent. Nonetheless, when analysing a social network a longer time period is advantageous. However, for our purposes of demonstrating our method, 28 days is sufficient.

Next we identified a subset consisting 7 241 148 of the Tweets that contained a mention of another account (@name). These Tweets were sent by 260 439 accounts.

Finally, we selected the subset of accounts that mentioned any other of the accounts for which there was also a reciprocal mention. That is, those accounts, say Xname, that sent at least one Tweet containing a mention of another account, say Yname (a mention @Yname), where there is at least one reciprocal Tweet made by Yname containing a mention of Xname (a mention @Xname). This resulted in the selection of 59 789 accounts, with each having a reciprocated mention of at least one other account. Representing those accounts as vertices, and the reciprocated mentions as undirected, unweighted edges, we thus obtained a 10 city-based Twitter reciprocated mentions network defined for October 2014. It has average degree of 2.36.

The employment of reciprocated mentions (as opposed to say mutual following) shows that the accounts not only are aware of each other but have actively engaged with each other during the prescribed interval of time (28 days). In that sense the undirected network contains validated (active), two-way connections. The full dataset for this network is available in the electronic supplementary material, where the first column relates to the city (1: Edinburgh; 2: Glasgow; 3: Cardiff; 4: Bristol; 5: Nottingham; 6: Birmingham; 7: Sheffield; 8: Leeds; 9: Manchester and 10: London), and the other two columns indicate an edge between the two listed nodes.

Each of the accounts is associated with at least one city. In many cases, they may be associated with more than one city. For example, they might state ‘I am from Manchester, working in London’ implying they may be genuinely connected into more than one city-based social network. In fact out of the 59 789 accounts we have, 29 814, 16 935, 10 680, 6236, 3949, 5890, 6043, 5644, 4830 and 4061 accounts were associated with London, Manchester, Leeds, Sheffield, Birmingham, Nottingham, Cardiff, Bristol, Glasgow and Edinburgh, respectively. So on average each account claims to be associated with 1.57 cities.

We may restrict the mentions network to each separate city as follows. First, we examine the subnetwork containing those accounts associated with each city. We only considered accounts that are active within a particular city, so if an account has reciprocated mentions with another account within that city, it is included within the corresponding network. This results in 10 city-based reciprocated mentions networks. Each of the city-based mentions networks contains a single giant connected component and a very large number of smaller connected components (with less than a handful of vertices).

**Figure 2.**
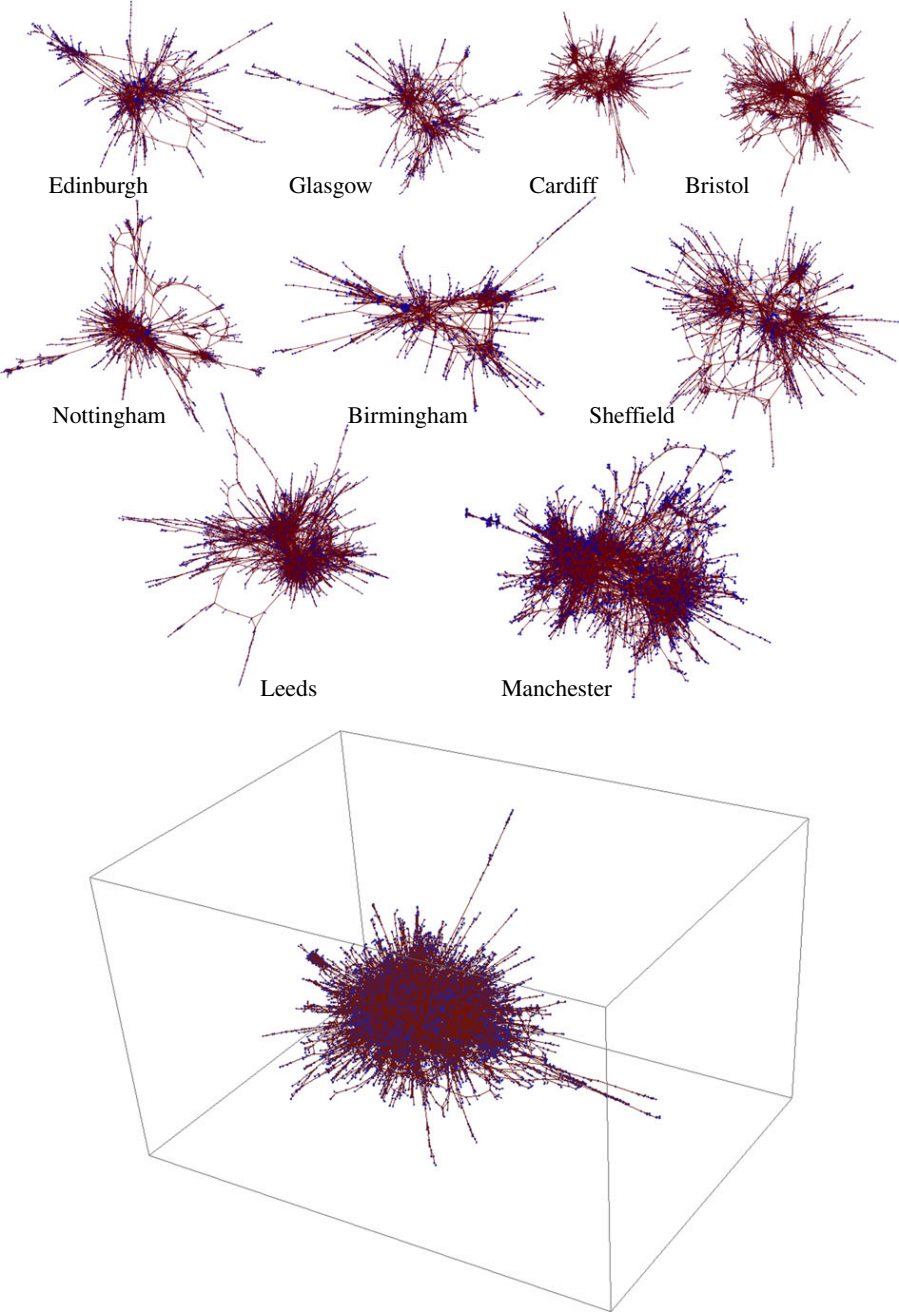
The giant component for each of the 10 cities drawn using spring-electrical embedding in two-dimensional space, except London which is drawn in three-dimensional space.

In this paper, we shall focus on these giant components as being representative of the nature of the digital social network for each city, as represented by the corresponding reciprocated Twitter mentions network. Details of the giant component for each city are described in table 1. Additionally, figure 2 shows the giant components drawn using spring-electrical embedding (GraphPlot in Mathematica, with the option Method → SpringElectricalEmbedding). The spring-electrical embedding algorithm assigns two forces between each pair of nodes, a local attractive force and a global ‘electrical’ force. The graph is drawn so as to minimize the overall energy of the system, where the energy is defined as a function of these two forces.

**Table 1.** Key information about the social network for each city: number of accounts, mean degree, degree variance, clustering coefficient, number of communities, modularity and the difference between modularity and the lower bound on modularity (from equation 2.4).

city	#accts	av. degree	degree var.	clust. coeff.	*R*	*Q*	diff.
Birmingham	1321	3.017	17.24	0.111	45	0.795	0.047
Edinburgh	1645	2.609	7.95	0.070	38	0.841	0.027
Glasgow	1802	2.535	8.49	0.065	39	0.865	0.044
Nottingham	2066	3.054	18.71	0.119	55	0.827	0.068
Cardiff	2685	3.310	21.24	0.196	44	0.859	0.083
Sheffield	2845	3.092	16.19	0.128	52	0.855	0.088
Bristol	2892	3.138	18.30	0.107	74	0.803	0.019
Leeds	5263	3.541	53.38	0.101	133	0.735	0.015
Manchester	7646	3.182	29.14	0.072	145	0.820	0.037
London	16 171	3.001	20.64	0.097	156	0.869	0.159

Each city may be partitioned into separate communities by the optimization of modularity. In table 1, we show the corresponding optimal number of communities and the corresponding value for *Q*. These values are relatively high compared with the modularities of random graphs conditioned solely on the corresponding degree distributions. In line with intuition, London is exceptional. The lower bound for modularity (equation (2.4)) is considerably weaker than the lower bounds for other cities. Nonetheless, it is a tight bound, especially for Bristol and Leeds. This implies that London has many connecting edges joining communities, whereas Bristol and Leeds do not.

There is a number of heuristic methodologies that have been designed to produce the optimal partition (and thus the modularity, *Q*) [15–17]. In all of the calculations here we have used a standard method based on the successive bisection of communities. Starting out from the whole network (a trivial one subset partition), this method successively identifies a bisection of any one of the subsets within the present partition that produces the maximal increase to the overall modularity. It halts when no further bisections of any subset within the present partition improves the overall modularity. This method is implemented in Mathematica (FindGraphCommunities, with the option Method → Modularity).

**Figure 3.**
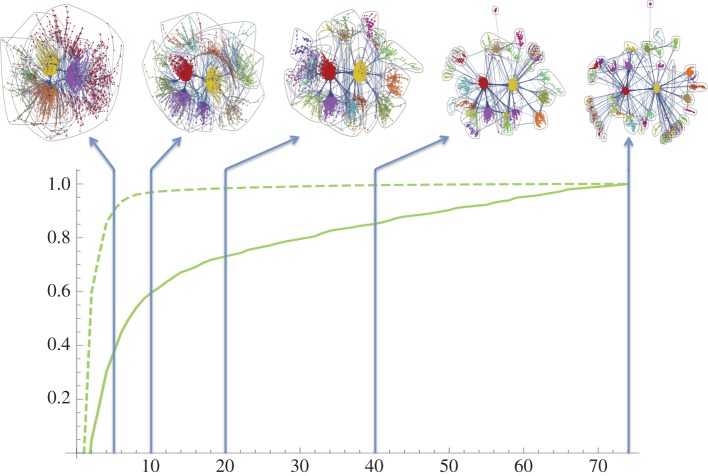
This shows the Bristol giant component being successively sub-divided into communities (*n* = 2892 and *R* = 74). We plot the relative modularity (dashed line) and the relative Shannon entropy (solid line) as a function of *k* for *k*=1,…,*R*.

As an example in figure 3, we show the Bristol giant component becoming partitioned as the number of communities increases up to *R*=74. We show both the modularity and the Shannon partition entropy (equal to $-\sum_{j=1}^{k}p_{j}\log p_{j}$, where *p*_*j*_ denotes the fraction of vertices present within the *j*th subset of the optimal *k*-wise partition) as a fraction of their optimal values obtained at 74 communities. The behaviour is very similar to that observed in Onnela *et al.* [17], where an alternative, spectral, approach to optimizing *Q* was taken while introducing a degree of freedom to control the relative scale of discrimination (equivalent here to controlling the number of communities sought, *k*=1,…,*R*). The modularity is almost optimal when *k*=20 or so, with the remaining work, in splitting off smaller communities, representing some fine-tuning. Since modularity is not very sensitive, assuming equality within equations (2.1) and (2.2) is justified.

## 5. Decomposing real networks and constructing virtual networks

Consider the decomposition of the social network of a city, called City *A*, such as Bristol shown in figure 3. We may use the modularity calculation to identify the component sub-communities of a city, and consider the set of components as a sample from the underlying distribution that makes up cities *like* City *A*.

The idea employed here is very similar to that of bootstrapping [18]. We have a sampled set, *X*, containing sub-communities (observed within City *A*). If we resample each one of them exactly once and then take the union we arrive at a city very similar to City *A* itself, that is just missing the relatively small number of edges connecting across sub-communities (we could compensate for this minor diversion from reality by adding some edges at random). Now let us resample from *X*, independently with repetition for as many times, *R*, as we wish. For each value of *R*, we may obtain an ensemble of virtual cities (an ensemble where each such city contains exactly *R* sub-communities), that are made up of the same types and distribution of communities as we observed in City *A*. As with bootstrapping, we use the observed distribution, *X*, to represent our best sampled estimate for the underlying (and unobserved) distribution $\tilde{X}$ of sub-communities from which cities like *A* are composed.

Of course, if City *A* has very few identifiable communities (a low resolution partition) forming up *X*, then it will be a poor representation of the underlying possibilities in $\tilde{X}$. As the number of distinct sub-communities in *X* increases, we may see much more diverse behaviour inherited from $\tilde{X}$, that is a higher variance.

Suppose that we independently resample exactly *R* sub-communities from *X* and take their union to form-up a new city. The *s*th sub-community that we sample has associated observables (*Qs*,*F*_*s*_,*m*_*s*_,*n*_*s*_,…). Let $\left({\bar{n}}_{X},{\bar{m}}_{X}\right)$ denote the mean numbers of vertices and edges for all sub-communities in *X*. Then

$$\sum_{s=1}^{R}n_{s}\to R\;{\bar{n}}_{X}\;\mathrm{and}\;\sum_{s=1}^{R}m_{s}\to R\;{\bar{m}}_{X},$$

and similarly for any other quantities.

From (2.2), assuming that lower bound is valid as an estimate (which we have already observed as reasonable in experiments), we have

5.1 $$Q∼\frac{1}{R}\frac{{\bar{\left(m_{s}^{2}Q_{s}\right)}}_{X}}{{\bar{m_{s}}}_{X}^{2}}+\frac{{\bar{F_{s}}}_{X}}{2{\bar{m_{s}}}_{X}}-\frac{1}{R}\frac{{\bar{\left(m_{s}F_{s}\right)}}_{X}}{2{\bar{m_{s}}}_{X}^{2}}.$$

Now if we select *R*=|*X*| then we are constructing virtual cities of a similar size to the conditioning City *A*, and we may create an ensemble of such virtual cities that will include the conditioning city. Now consider another city, say City *B*, that is observed to have *n*_*B*_ vertices. Set $R\approx n_{B}/{\bar{n}}_{sX}$. Then making *R* draws from *X*, randomly with replacement, we may construct an ensemble of virtual cities that are all approximately the same size as City *B*, yet that are made up of components from the same distribution, *X*, as City *A*. Thus, the deviation or otherwise of measures for *B*, such as *Q*, from the distribution for the same measure obtained from this virtual ensemble can indicate whether or not it is reasonable to assume that *B* may be made up of the same distribution of sub-communities as *A*.

**Figure 4.**
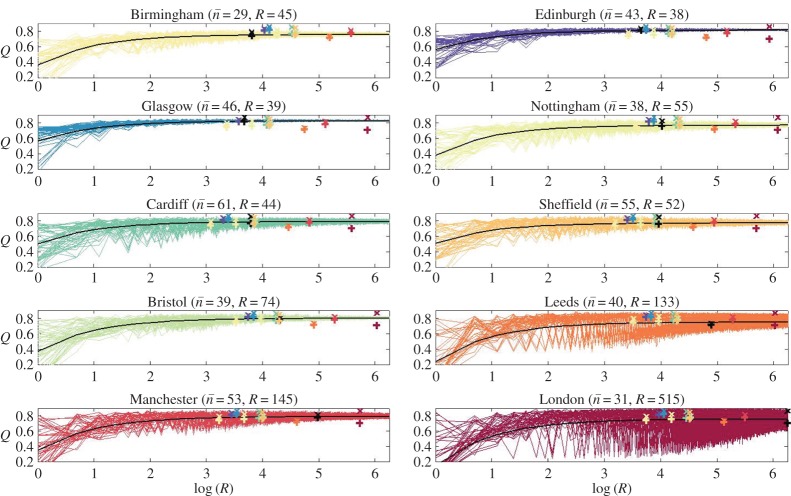
Ensembles of modularities, *Q*, for virtual cities, as ln⁡(R) varies, made from elements resampled from *X*, the set of sub-communities from the conditioning city, labelled above each plot. Crosses show the actual (ln⁡(R),Q) pairs for the observed cities. Pluses show the lower bounds on *Q* obtained from each city’s optimal partition and using equation (2.2) to estimate their *Q*-value (assuming a union of their separated communities, in the spirit of the virtual cities within the ensembles). The black crosses and pluses relate to the conditioning city. The coloured crosses relate to the various other cities, whose colour corresponds to the plot for that city as the conditioning city.

In figure 4, we carry out this programme where a city is employed as City *A*. On top of the ensemble envelope of *Q*-values obtained for all of the virtual cities we show the other observed cities. The size of the envelope, which is generally proportional to the number of communities *R*, is a guide as to the variety of communities within a city. For example, Glasgow and Edinburgh have small envelopes indicating fairly homogeneous communities. Therefore, a city with a wider envelope, such as Manchester, contains social structures not seen in Glasgow and Edinburgh.

Consider Bristol as the conditioning city. It is clear that while Manchester, Nottingham, Edinburgh and Birmingham are within the equivalent Bristol-like envelope, Leeds, especially, is not. We would contend that the social structure observed within Leeds is not consistent with that observed within Bristol; even when we allow for changes of scale, there must be some specific elements that are present or absent on one or another. Thus, the performance of any interventions or other activities designed to work within such social networks cannot be extrapolated from Bristol to Leeds. However, since Leeds contains a wider variety of social structures, a Bristol-like city could be created from Leeds communities. The transitive properties from one city to another, excluding London, are summed up in table 2.

**Table 2.** Communities from cities on the left can be used to replicate cities indicated by a tick.

communities from	Bi.	Ed.	Gl.	No.	Ca.	Sh.	Br.	Le.	Ma.
Birmingham							✓	✓	
Edinburgh	✓			✓			✓		✓
Glasgow	✓	✓		✓					✓
Nottingham	✓	✓					✓	✓	
Cardiff	✓	✓		✓			✓	✓	✓
Sheffield	✓	✓	✓	✓	✓		✓	✓	✓
Bristol	✓	✓		✓					✓
Leeds	✓	✓	✓	✓	✓	✓	✓		✓
Manchester	✓	✓	✓	✓	✓	✓	✓	✓	

## 6. Discussion

We have presented a way to estimate the approximate maximum modularity of a large network based on the maximum modularities of the communities within the network (independent of the method used to define communities).The lower bound estimate presented here is useful because modularity lacks sensitivity near the optimal resolution. Maximizing modularity is an *NP* hard problem. Therefore, representing the modularity of a graph in terms of the modularity of the communities is computationally advantageous.

We presented the null model where we built a large city from ghettos represented as a combination of Erdos–Rényi graphs. We showed that the modularity converges after the city had been divided into approximately 20 communities, irrespective of the total number of communities.

We represented cities as combinations of communities from different cities, the ‘conditioning city’. This showed many cities, such as Bristol, Nottingham, Edinburgh, Manchester and Birmingham, comprise communities with similar structure—despite their size and location varying considerably. Conversely, it also identified cities like Leeds, which are not made up of similar communities to other cities we sampled. Leeds, despite being smaller than Manchester, contains social structures not observed in Manchester. At the other end of the variance spectrum, Edinburgh and Glasgow have very little variance between their communities. Therefore, recreating cities with greater community variance is challenging from these limited pools of Scottish communities. This type of analysis can aid decisions about social media activities and interventions. For example, a campaign that is successful in Bristol should also be successful in Manchester (but not Leeds). Since Bristol is considerably smaller, it may prove useful to test the campaign in Bristol first.

The method is not limited to Twitter data. One could apply the same method to any form of network that could define a city, such as Facebook or e-mail. Furthermore, we are not restricted to cities. It would be interesting to compare universities to each other, or countries, or the social structure in different governments. The method is only limited by our imaginations and the data available.

## Supplementary Material

Networks for 10 different cities from Twitter data from reciprocated tweets during October 1st 2014 through to October 28th 2014 (four full weeks). The first column is the city, second is a node i, which is connected to node j, the third column. 1. Edinburgh; 2. Glasgow; 3. Cardiff; 4. Bristol, 5. Nottingham; 6. Birmingham; 7. Sheffield; 8. Leeds; 9. Manchester; 10. London. *** THE WHOLE FILE IS NOT UPLOADING. CURRENTLY ONLY CITIES 1 TO 5 ONLY ARE UPLOADED ****"

## Data accessibility

Data can be accessed at http://dx.doi.org/10.5061/dryad.2gf23.

## Appendix A. Solutions to equation (2.5)

We seek solutions of the aggregating functional relationship

A 1 $$H(Q,R_{a}R_{b})=H(H(Q,R_{a}),R_{b}),$$

where *H*(*Q*,1)=*H*(*Q*). Immediately, we may see that

A 2 $$H(Q,R_{a})=\frac{Q}{{\left(R_{a}\right)}^{\alpha }}$$

is a solution for all *α*∈ℛ, *α*≠0. Consider the anzatz

A 3 $$H(Q,R_{a})=\frac{Q}{{\left(R_{a}\right)}^{\alpha }}+g(R_{a}),$$

where *g*(1)=0. Then *g* must satisfy

A 4 $$\frac{g(R_{a})}{{\left(R_{b}\right)}^{\alpha }}+g(R_{b})=g(R_{a}R_{b}).$$

Differentiating with respect to *R*_*a*_, we obtain

A 5 $$\frac{g^{\prime }(R_{a})}{{\left(R_{b}\right)}^{\alpha }}=g^{\prime }(R_{a}R_{b})R_{b}.$$

Now setting *R*_*a*_=1 and *g*′(1)=*αβ*, for some β∈R, we see that *g* must satisfy

A 6 $$g^{\prime }(R_{b})=\frac{\alpha \beta }{{\left(R_{b}\right)}^{\alpha +1}},$$

and *g*(1)=0. Hence *g*(*R*_*b*_)=*β*−*β*(*R*_*b*_)^−*α*^, and thus

A 7 $$H(Q,R_{a})=\frac{Q}{{\left(R_{a}\right)}^{\alpha }}+\beta \left(1-\frac{1}{{\left(R_{a}\right)}^{\alpha }}\right).$$
