# Correction to ‘Comparison of social structures within cities of very different sizes’

**DOI:** 10.1098/rsos.160243

**Published:** 2016-05-04

**Authors:** P. Grindrod, T. Lee

*R. Soc. open. sci.* **3**, 150526. (2016; Published 24 February 2016). (doi:10.1098/rsos.150526)

**Amendment to table 2**

**Table 2.** Communities from cities on the left can be used to replicate cities indicated by a tick.

communities from	Bi.	Ed.	Gl.	No.	Ca.	Sh.	Br.	Le.	Ma.
Birmingham				✓	✓	✓	✓		✓
Edinburgh			✓						
Glasgow		✓							
Nottingham		✓	✓		✓	✓	✓		✓
Cardiff	✓	✓	✓	✓		✓	✓		✓
Sheffield	✓	✓	✓	✓	✓		✓	✓	✓
Bristol	✓	✓	✓	✓	✓	✓			✓
Leeds	✓	✓	✓	✓	✓	✓	✓		✓
Manchester	✓	✓	✓	✓	✓	✓	✓	✓	

The first few rows are amended so that the table is now in line with figure 4 and corresponds to the text. Additionally, it highlights that Nottingham, Cardiff, Sheffield, Bristol and Manchester are alike cities.

